# Differences and biocontrol potential of haustorial endophytic fungi from *Taxillus Chinensis* on different host plants

**DOI:** 10.1186/s12866-023-02878-x

**Published:** 2023-05-13

**Authors:** Li-sha Song, Juan Huo, Lingyun Wan, Limei Pan, Ni Jiang, Jine Fu, Shugen Wei, Lili He

**Affiliations:** 1 Guangxi Botanical Garden of Medicinal Plants, Nanning, 530023 China; 2 Guangxi Key Laboratory for High-Quality Formation and Utilization of Dao-di Herbs, Nanning, China

**Keywords:** Plant endophytes, Inhibitory activity, Biodiversity, Phylogeny, *Taxillus chinensis*

## Abstract

**Background:**

To explore the community composition and diversity of the endophytic fungi in *Taxillus chinensis*, samples of the parasites growing on seven different hosts, *Morus alba*, *Prunus salicina*, *Phellodendron chinense*, *Bauhinia purpurea*, *Dalbergia odorifera*, *Diospyros kaki* and *Dimocarpus longan*, were isolated. The strains were identified by their morphological characteristics and their internal transcribed spacer (ITS) sequences.

**Results:**

150 different endophytic fungi were isolated from the haustorial roots of the seven hosts with a total isolation rate of 61.24%. These endophytic fungi were found to belong to 1 phylum, 2 classes, 7 orders, 9 families, 11 genera and 8 species. Among of them, *Pestalotiopsis*, *Neopestalotiopsis* and *Diaporthe* were the dominant genera, accounting for 26.67, 17.33 and 31.33% of the total number of strains, respectively. Diversity and similarity analyses showed that the endophytic fungi isolated from *D. longan* (H’=1.60) had the highest diversity index. The highest richness indexes were found in *M. alba* and *D. odorifera* (both 2.23). The evenness index of *D. longan* was the highest (0.82). The similarity coefficient of *D. odorifera* was the most similar to *D. longan* and *M. alba* (33.33%), while the similarity coefficient of *P. chinense* was the lowest (7.69%) with *M. alba* and *D. odorifera*. Nine strains showed antimicrobial activities. Among them, *Pestalotiopsis sp., N. parvum* and *H. investiens* showed significant antifungal activity against three fungal phytopathogens of medicinal plants. At the same time, the crude extracts from the metabolites of the three endophytic fungi had strong inhibitory effects on the three pathogens. *Pestalotiopsis sp., N. parvum* and *H. investiens* had the strongest inhibitory effects of *S. cucurbitacearum*, with inhibitory rates of 100%, 100% and 81.51%, respectively. In addition, *N. parvum* had a strong inhibitory effect on *D. glomerata* and *C. cassicola*, with inhibitory rates of 82.35% and 72.80%, respectively.

**Conclusions:**

These results indicate that the species composition and diversity of endophytic fungi in the branches of *T. chinensis* were varied in the different hosts and showed good antimicrobial potential in the control of plant pathogens.

**Supplementary Information:**

The online version contains supplementary material available at 10.1186/s12866-023-02878-x.

## Background

Endophytic fungi are fungi that live in plant tissues and have no obvious disease symptoms at a certain period of their life history. They generally colonize healthy plant tissues and organs with a wide variety of species and wide distribution [1–3]. Since Stierle et al. first reported that an endophytic fungus isolated from *Taxus breviflia* was able to synthesize paclitaxel, an anticancer substance, the study of such fungi in medicinal plants has become a research hotspot [4]. It was reported that there were significant differences in the diversity, community structure and composition of endophytic fungi in leaves, with differences in the community structure increasing with the age of the fungi present [5]. For example, the dominant fungi in the endophytic fungal community were *Aspergillus*, *Candida* and *Mycosphaerella* [5]. Of these, the diversity of endophytic fungi isolated from the tubers was the highest.

Liu et al. found that the community diversity of endophytic fungi in *Astragalus membranaceus* was low, and *Hemileia* and *Gibberella* were the dominant populations of these fungi [6]. Yang et al. found that *Lophiostoma* was the dominant genus in the community composition of endophytic fungi in the bark of *Eucommia ulmoides* from three habitats [7]. The population structure and distribution patterns of endophytic fungi is closely related to environmental changes, such as temperature, humidity, light, geographical location and the surrounding vegetation, classification of host plants and genetic background [8, 9]. In addition, the growth stage configuration (age) of host plants and tissues may also affect the species composition of endophytic communities [10]. Only a few specific endophytic fungi can be colonized in these plants, resulting in a certain regional specificity of the endophytic fungi population structure [11]. Different endophytic species have been found in the parenchyma and vascular tissues of host plants of different ages [12].

Recent studies have shown that endophytic fungi with the ability to produce active substances that inhibit pathogenic fungi can be isolated and screened from different tissues and organs of healthy plants [13–18]. For example, the organic crude extracts isolated from *Trichoderma erinaceum* significantly inhibited the mycelial growth of *Pythium ultimum* [19]. In addition, *T. harzianum* can be used as a biological control agent to inhibit post-harvest avocado (*Persea americana* Mill) pathogens, thereby avoiding significant losses of this internationally important fruit [20]. Bioactive compounds exclusively produced by these endophytic fungi are important for improving their adaptability to host plants and increasing their tolerance to biological and abiotic stresses. In addition, these compounds can induce the production of a large number of known and novel bioactive secondary metabolites [21–23]. These can not only promote seedling growth and improve seedling resistance to drought, disease, predatory insects and non-physiological salt concentrations, but also improve the medicinal efficacy of host plants, which plays an important role in their medicinal value [22–30].

*Taxillus chinensis* belonging to the Loranthaceae family are mainly distributed in the southern and southwestern areas of China. The dried stems and leafy branches of *T. chinensis* are commonly used as materials for traditional Chinese medicine and are known as “Sang Ji Sheng” in China. *T. chinensis* has a high medicinal value. It is used to relieve rheumatism, to strengthen the liver and kidney, the muscles and bones, as well as to prevent spontaneous abortions [31]. Meanwhile, *T. chinensis* is also used as raw materials for making parasitism tea in China, and it is exported to nearly 30 countries in Southeast Asia [32]. While there is an increasing market demand for this product, it also has a very important position in Guangxi and it is even sold on the national Chinese herbal medicine markets.

To date, there are only a few reports to investigate biodiversity of endophytic fungi of the genus *Pestalotiopsis*. Gong et al. isolated an endophytic fungus of the genus *Pestalotiopsis* from the *T. chinensis*, which had significant inhibitory effects on cultured A549 and H460 tumor cells [33]. Therefore, in this study, seven host species, including *Morus alba*, *Prunus salicina*, *Phellodendron chinense*, *Dalbergia odorifera*, *Bauhinia purpurea*, *Diospyros kaki* and *Dimocarpus longan* were selected as branches of *T. chinensis*, respectively. They were used to survey the endophytic fungi diversity of *T. chinensis* on different hosts of this species. We also evaluated their antagonistic effects against three important plant pathogens, *Corynespora cassicola* of *Sarcandra glabra*, *Didymella glomerata* of *Sophora tonkinensis* and *Stagonosporopsis cucurbitacearum* of *Siraitia grosvenorii*. The endophytic fungi strains with strong antifungal activity were screened out to lay a foundation for the development of new antimicrobial resources. To the best of our knowledge, this is the first time the diversity and antifungal activity of endophytic fungi in *T. chinensis* were studied systematically.

## Results

### IR of endophytic fungi

**Fig. 1:**
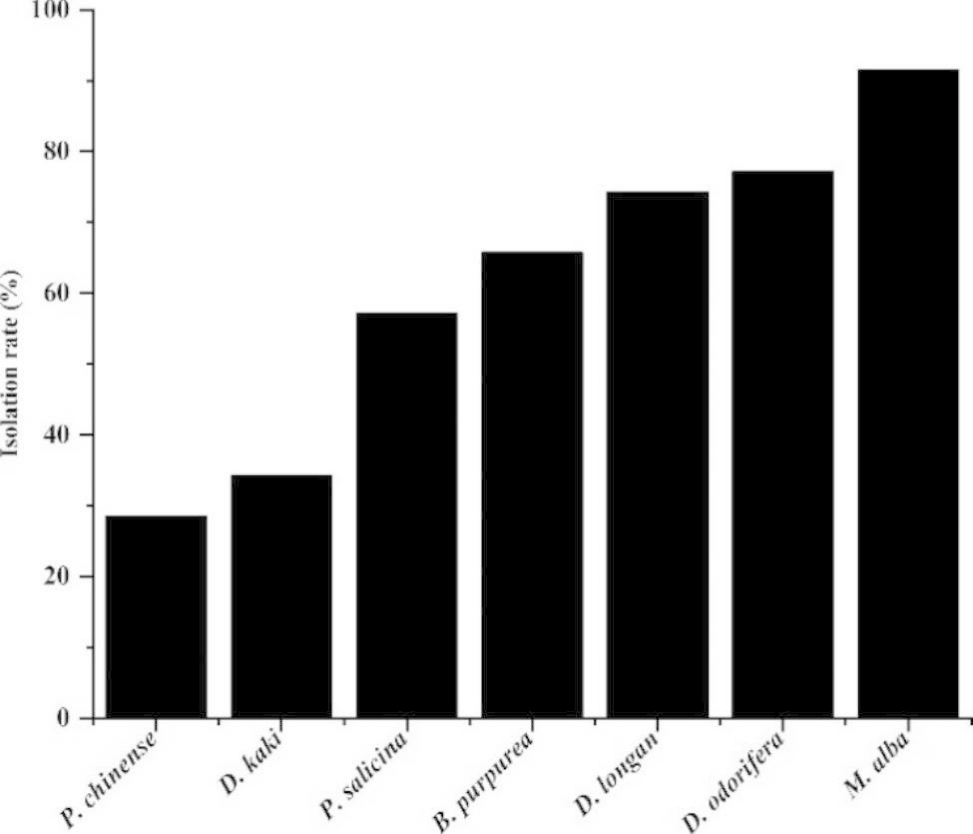
The isolation rates of endophytic fungi from *T. chinensis*

A total of 150 endophytic fungal strains were isolated and purified from 245 tissue segments from *T. chinensis* parasitized on different host plants and the average IR of the endophytic fungi from *T. chinensis* was 61.22%. However, the IR of endophytic fungi from *T. chinensis* varied greatly due to the differences of host plants species. The highest IR of endophytic fungi was obtained from *T. chinensis* parasitized on *M. alba*, and this was 91.43%. However, the lowest IR of endophytic fungi was obtained from *T. chinensis* parasitized on *P. chinense* and this was only 28.57% (Fig. 1 and Table S1).

### Colony composition of endophytic fungi of *T. chinensis*

**Fig. 2:**
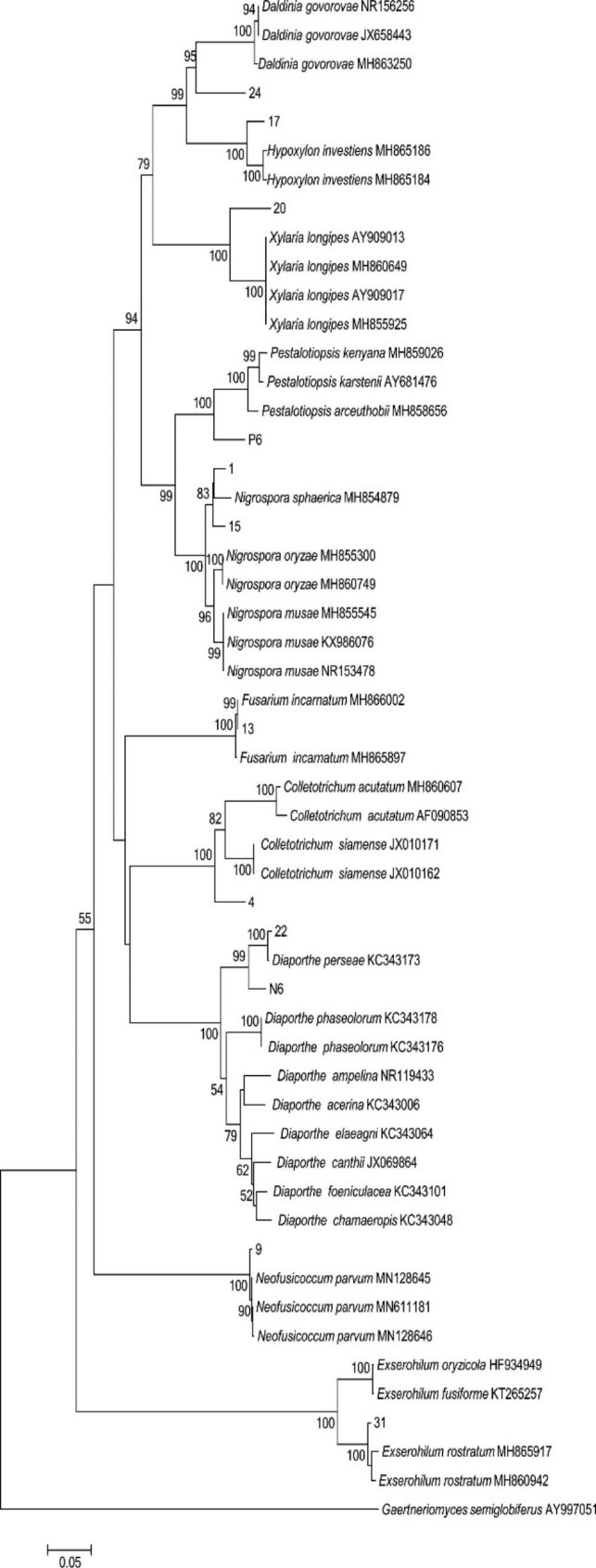
Phylogenetic analysis of endophytic fungi from *T. chinensis* parasitized on different host plants based on rDNA-ITS.

**Fig. 3:**
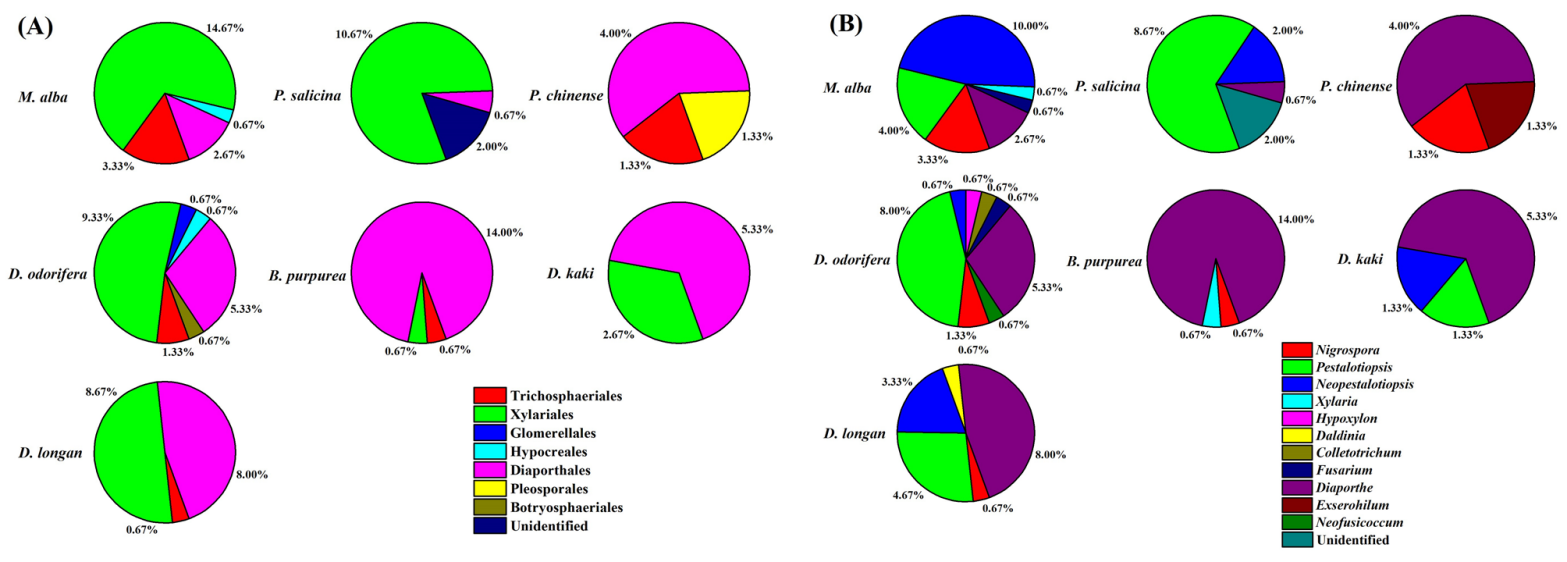
The isolation frequency of orders (**A**) and genera (**B**) related to *Taxillus chinensis* parasitized on different host plants

The morphological characteristics and molecular biology of the isolated strains were combined to determine the classification of the endophytic fungi in this study. There were 150 strains of endophytic fungi as well as three strains which were not clearly identified. The overall IR was 61.24% (Table 1). Other strains belonged to 1 phylum, 2 classes, 7 orders, 9 families, 11 genera and 12 species (Fig. 2), including ascomycetes, Ascomycota. Sordariomycetes was the dominant group, accounting for 96.00% of the total number of strains. At the order level, the dominant one was Xylariales, accounting for 46.67% of the total number of strains (Fig. 3-A and Table s2). At the genus level, *Pestalotiopsis*, *Neopestalotiopsis* and *Diaporthe* were the dominant genera, accounting for 26.67, 17.33 and 31.33% of the total strains, respectively (Fig. 3-B). *Nigrospora*, *Xylaria*, *Fusarium* and *Exserohilum* were the common genera, accounting for 7.33, 1.33, 1.33, 1.33% of the total strains, respectively (Fig. 3-B). *Hypoxylon*, *Daldinia*, *Colletotrichum* and *Neofusicoccum* were rare genera, accounting for 0.67% of the total strains (Table 1; Fig. 3-B and Table s3). Sequences of these twelve strains were submitted to the GenBank database, and the accession numbers obtained were MZ836840, MZ823600, MZ2823598, MZ836841, MZ836842, MZ836843, MZ823601, MZ836844, MZ823599, MZ836845, MZ823597 and MZ836846 for strains 1, 15, P6, 20, 17, 24, 4, 13, N6, 22, 31 and 9, respectively.

**Table 1:** Community composition of the endophytic fungi of different hosts of Taxillus chinensis

Phylum	Class	Order	Family	Genus	Species	N	IF (%)
Ascomycota	Sordariomycetes	Trichosphaeriales	Trichosphaeriaceae	*Nigrospora*	*Nigrospora* spp.	10	6.67
					*Nigrospora sphaerica*	1	0.67
		Xylariales	Sporocadaceae	*Pestalotiopsis*	*Pestalotiopsis* spp.	40	26.66
				*Neopestalotiopsis*	*Neopestalotiopsis* spp.	26	17.33
			Xylariaceae	*Xylaria*	*Xylaria longipes*	1	0.67
					*Xylaria* sp.	1	0.67
			Hypoxylaceae	*Hypoxylon*	*Hypoxylon investiens*	1	0.67
				*Daldinia*	*Daldinia govorovae*	1	0.67
		Glomerellales	Glomerellaceae	*Colletotrichum*	*Colletotrichum* sp.	1	0.67
		Hypocreales	Nectriaceae	*Fusarium*	*Fusarium incarnatum*	1	0.67
					*Fusarium* sp.	1	0.67
		Diaporthales	Diaporthaceae	*Diaporthe*	*Diaporthe* spp.	58	38.66
					*Diaporthe perseae*	2	1.33
	Dothideomycetes	Pleosporales	Pleosporaceae	*Exserohilum*	*Exserohilum rostratum*	2	1.33
		Botryosphaeriales	Botryosphaeriaceae	*Neofusicoccum*	*Neofusicoccum parvum*	1	0.67
	Unclassidied fungi			Unidentified		3	2.00
Total						150	100

Note: N is the number of different strains; IF is the isolation frequency

### IR of endophytic fungi in different hosts of *T. chinensis*

The number of endophytic fungal strains isolated from *T. chinensis* branches of different hosts was different, with the highest number of 32 endophytic fungal strains isolated from *M. alba.* This was followed by *D. odorifera*, *D. longan*, *B. purpurea*, *P. salicina* and *D. Kaki* which consisted of 27, 26, 23, 20 and 12 strains, respectively. Only 10 were isolated from *P. chinense*.

**Fig. 4:**
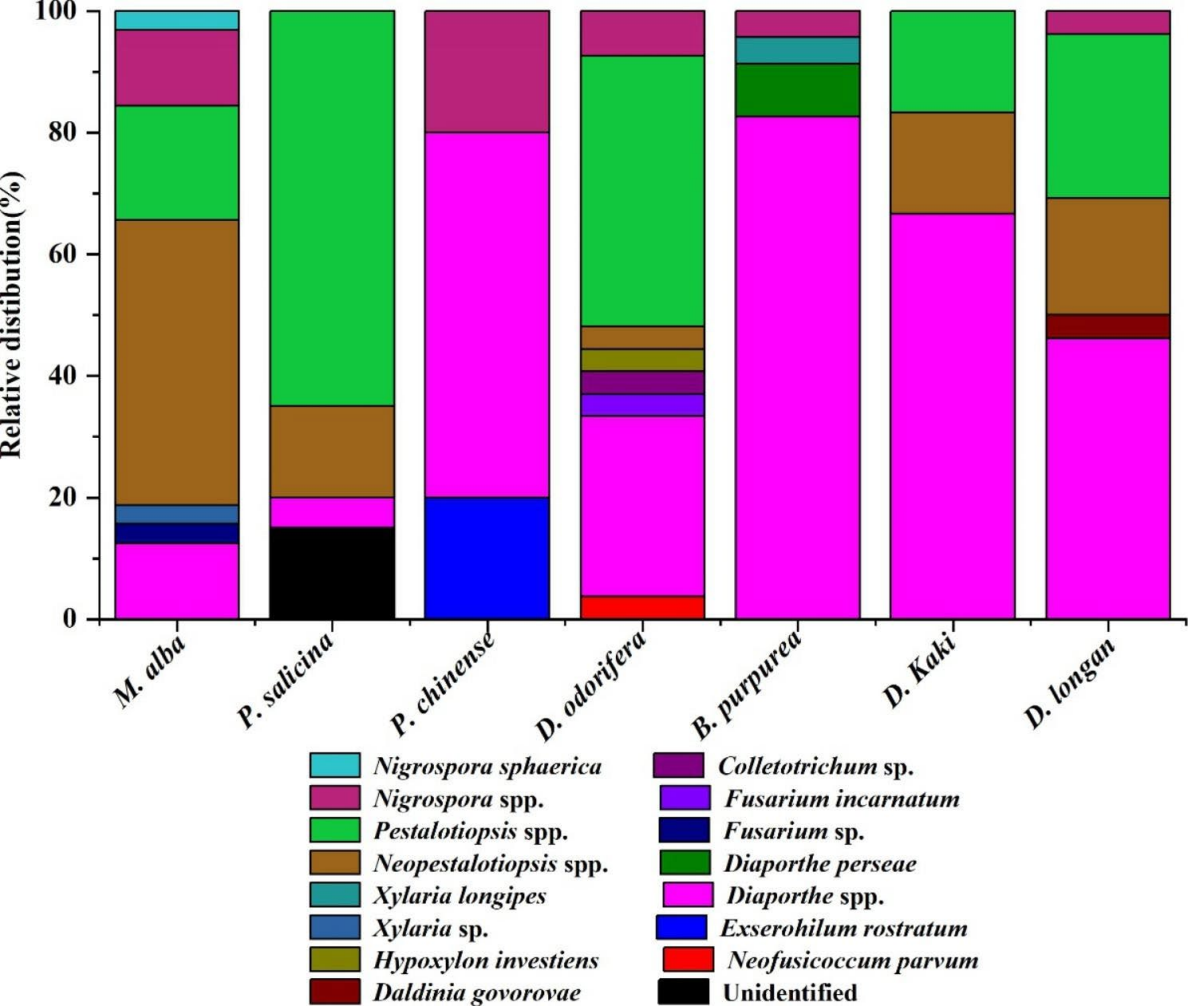
The diversity and distribution of endophytic fungi isolated from *Taxillus chinensis* parasitized on different host plants

The IFs of endophytic fungi from the branches of *T. chinensis* of the seven hosts were also analyzed. The most frequently isolated endophytic fungi were *Pestalotiopsis* and *Neopestalotiopsis* in the parasitic branches of *T. chinensis* from *M. Alba*, *D. odorifera*, *D. Longan*, *P. salicina* and *D. Kaki*. Among them, *M. alba* and *D. odorifera* had the highest separation frequencies with both reaching 7.33%. In addition to *P. chinense*, the endophytic fungi with the highest IF in the parasitic branches of the other 6 hosts were *Diaporthe*, among which *D. odorifera* had the highest IF of up to 12%, followed by *B. purpurea* which was up to 10% (Fig. 4 and Table s4).

### Diversity of endophytic fungi of *T. chinensis* in different hosts

Table 2 shows the diversity index of endophytic fungi of the haustoria of *T. chinense* that parasitized the seven plants, *D. longan* (1.60) > *D. odorifera*(1.51) > *M. alba* (1.41) > *P. chinense* (1.16) > *B. purpurea* (1.03) > *P. salicina* > (0.86) > *D. kaki* (0.77). The richness indexes were calculated to be *M. alba* (2.23) = *P. chinense* (2.23) > *D. longan* (1.97) > *D. odorifera* (1.95) > *B. purpurea* > (1.29) > *D. kaki* (1.11) > *P. salicina* (0.69). The evenness indexes were *D. longan* (0.82) > *P. salicina* (0.79) > *D. odorifera* (0.73) > *P. chinense* (0.72) > *M. alba* (0.68) > *B. purpurea* (0.64) > *D. kaki* (0.56). The results showed that *D. longan* had the largest diversity index (1.60), with the largest richness index (2.23) of endophytic fungi of the haustoria found in *M. alba* and *P. chinense*. *D. longan* also had the largest evenness index (0.82).

**Table 2:** The diversity indexes of endophytic fungi of different hosts of *T. chinensis*

Host	Shannon-Weiner	Margalef’s	Evenness
	diversity index (H’)	index (D)	index
*M. alba*	1.41	2.23	0.68
*P. salicina*	0.86	0.69	0.79
*P. chinense*	1.16	2.23	0.72
*D. odorifera*	1.51	1.95	0.73
*B. purpurea*	1.03	1.29	0.64
*D. kaki*	0.77	1.11	0.56
*D. longan*	1.60	1.97	0.82

### Similarity coefficients of endophytic fungi of *T. chinensis* in different hosts

Table 3 shows that the similarity coefficients of endophytic fungi of the haustoria of *T. chinense* that parasitized different hosts were different. The similarity coefficient between *D. longan* and *D. kaki* was the highest, which was 36.36%. The similarity coefficient of *D. longan*, *M. alba* and *D. odorifera* was 33.33% and that of *P. salicina* and *D. kaki* was 28.57%. The similarity coefficient of *D. longan* and *M. alba* was 26.67%. The similarity coefficient was the lowest with *M. alba* and *P. chinense* with *D. odorifera* (7.69%), while there was no similarity coefficient with *P. salicina* and *D. kaki*, indicating that there were no similar endophytic fungi of the haustoria of *T. chinense* between *P. salicina* and *D. kaki* with *P. salicina*.

**Table 3:** The similarity coefficients of endophytic fungi of different hosts of *T. chinensis* (%)

Hosts	*M. alba*	*P. salicina*	*P. chinense*	*D. odorifera*	*B. purpurea*	*D. kaki*	*D. longan*
*M. alba*							
*P. salicina*	18.18						
*P. chinense*	7.69	0					
*D. odorifera*	33.33	18.18	7.69				
*B. purpurea*	15.38	12.5	10	23.08			
*D. kaki*	25	28.57	0	25	22.22		
*D. longan*	26.67	20	17	33.33	25	36.36	

### In vitro antagonistic assays of endophytic fungi against phytopathogens

The antagonistic activity of 9 fungal endophytes against the plant pathogens *C. cassicola*, *D. glomerate* and *S. cucurbitacearum* was evaluated in co-culture tests (Table 4). These 9 strains of endophytic fungi had different degrees of inhibition on the three pathogens, and the inhibition rates were above 60%. Among the 11 strains, *Neofusicoccum parvum* showed a significant antagonistic activity against *D. glomerata* and *C. cassicola*, and the inhibition rates were 85.29% and 78.82%, respectively. However, there was no inhibition with *S. cucurbitacearum*. The species, *Hypoxylon investiens*, was the one that had the best inhibition of *S. cucurbitacearum*. The inhibition rate was 83.53%.

**Table 4:** Inhibition of endophytic fungi on different pathogens found in medicinal plants

Strain	Species	Mean percentage of mycelial growth inhibition (%)		
		*D*. *glomerata*	*C. cassicola*	*S. cucurbitacearum*
4	*Colletotrichum* sp.	(68.82 ± 1.70)^a^	(60.00 ± 1.18)^a^	(69.41 ± 2.35)^a^
15	*Nigrospora sphaerica*	(70.59 ± 0.68)^ab^	(62.35 ± 1.18)^ab^	(62.35 ± 1.18)^b^
1	*Nigrospora* sp.	(71.76 ± 1.36)^ac^	(75.88 ± 1.76)^c^	(67.65 ± 0.59)^ac^
N6	*Diaporthe phaseolorum*	(73.53 ± 1.70)^bc^	(70.00 ± 4.12)^d^	(74.12 ± 1.18)^d^
P6	*Pestalotiopsis* sp.	(75.29 ± 2.03)^cd^	(71.18 ± 0.59)^de^	(75.88 ± 0.59)^de^
9	*Neofusicoccum parvum*	(85.29 ± 1.02)^e^	(78.82 ± 2.35)^cf.^	-
17	*Hypoxylon investiens*	(72.35 ± 0.34)^abcd^	(69.41 ± 1.18)^deg^	(83.53 ± 2.35)^f^
31	*Erostratum rostratum*	(74.71 ± 1.02)^cd^	(72.94 ± 1.18 )^cdegh^	(72.35 ± 2.94)^adg^
22	*Diaporthe perseae*	(78.82 ± 0.00)^df^	(62.94 ± 4.12 )^abi^	(75.29 ± 1.18)^degh^

Different lowercase letters in the same column represent a significant difference (P < 0.05)

### Antimicrobial activity of crude extracts from fermentation products of endophytic fungi

**Fig. 5:**
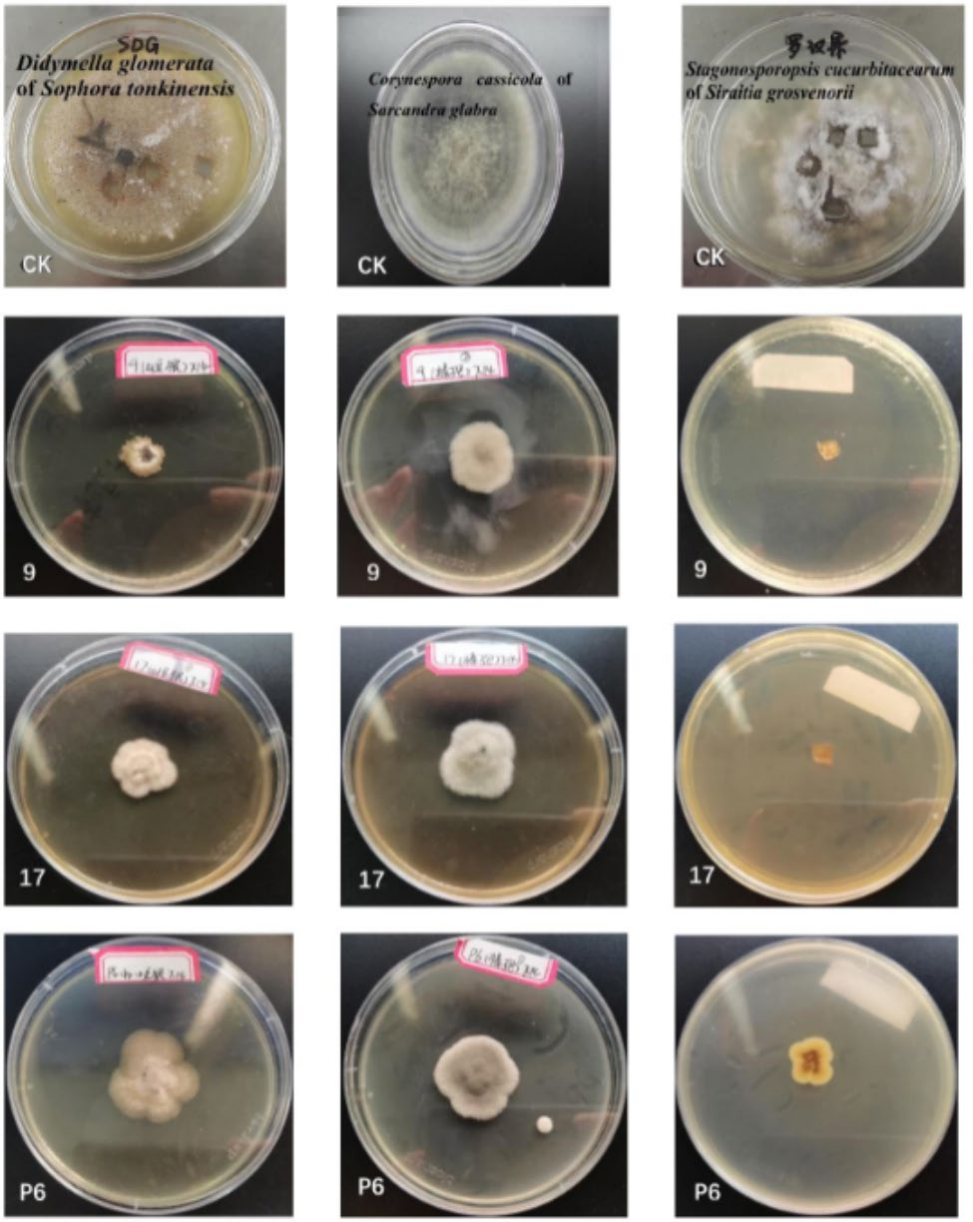
The inhibitory effects of crude extracts from fermentation products of endophytic fungi

**Fig. 6:**
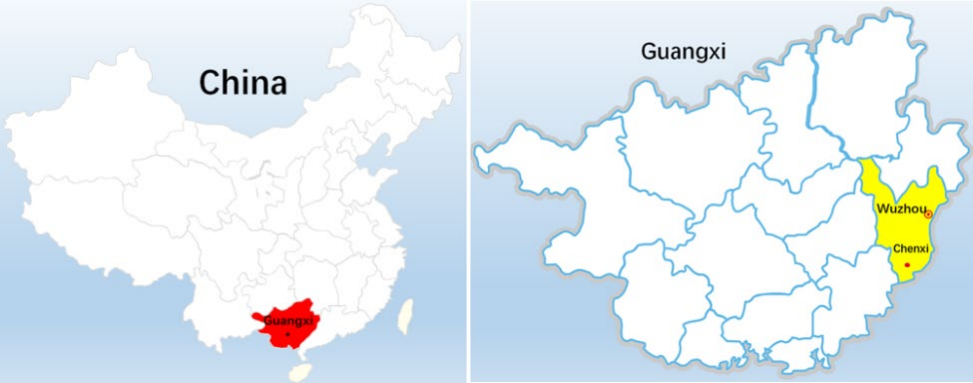
Maps showing the locations of the sample collection sites

As can be seen from Fig. 5 (Table s5), the crude metabolite extracts of the endophytic fungi strains 9, 17 and P6 of *T. Chinensis* seeds had obvious inhibitory effects on these three pathogens, and they can be used as fermentation production strains. The antifungal activities of the crude extracts of fermentation products of strains 9, 17 and P6 are shown in Fig. 6. As shown in Fig. 5 (Table s5), when the concentration of crude extracts was 100 mg/mL and they were cultured for 11 days, the results showed that they could significantly inhibit the growth of *D. glomerata*, *C. cassicola* and *S. cucurbitacearum*. Among these, strains 9, 17 and P6 had the strongest inhibitory effects of *S. cucurbitacearum* in Table 5, with inhibitory rates of 100, 100 and 81.51%, respectively. Secondly, strain 9 had strong inhibitory effects on *D. glomerata* and *C. cassicola*, with inhibitory rates of 82.35% and 72.80%, respectively. The extracts of strains 9, 17 and P6 were 6.456, 11.069 and 9.727 g, respectively.

**Table 5:** The antifungal activities of crude extracts from fermentation products of endophytic fungi

Strain	Species	Mean percentage of mycelial growth inhibition (%)		
		*D*. *glomerata*	*C. cassicola*	*S. cucurbitacearum*
9	*Neofusicoccum parvum*	(82.35 ± 1.22)^a^	(72.80 ± 3.21)^acd^	(100 ± 0)^g^
17	*Hypoxylon investiens*	(66.91 ± 0.85)^b^	(66.47 ± 0.34)^be^	(100 ± 0)^gh^
P6	*Pestalotiopsis* sp.	(77.45 ± 2.16)^ac^	(70.35 ± 3.29)^abcdf^	(81.51 ± 0.42)^aci^

## Discussion

In this study, a total of 150 endophytic fungi belonging to 11 genera were isolated from healthy haustorial roots of *T. chinensis* of 7 hosts from the same habitat in Guangxi, China. Among them, *Pestalotiopsis*, *Neopestalotiopsis* and *Diaporthe* had high IFs and were widely distributed in these hosts. As the dominant genus of endophytic fungi, these results are similar to those reported for endophytic fungi in *Taxilli herba* from *Salix babylonica* in Guangxi [33]. There were some differences in the distribution of endophytic fungi in the haustoria of *T. chinensis* of different hosts. Four species, *Exserohilum rostratum*, *Neofusicoccum parvum*, *Hypoxylon investiens* and *Nigrospora musae*, were isolated only from the haustorial roots of *P. chinense*, and *Daldinia govorovae* was just found from the haustorial roots of *M. longan*. Two species, *Xylaria longipes and Diaporthe perseae*, were exclusively isolated from the haustorial roots of *B. purpurea*, while *Nigrospora sphaerica, Xylaria* sp. and *Fusarium* sp. were isolated just from the haustorial branches of *T. chinensis*. In addition, *Colletotrichum acutatum and Fusarium incarnatum* were found only from the haustorial roots of *D. odorifera*. All these results indicated that the distribution of endophytic fungi had a certain host specificity [34–36]. When combined with the results of diversity and similarity coefficient analysis of endophytic fungi of the seven hosts, it was found that there were some differences among the endophytic fungi of the haustoria of *T. chinensis* in different plants. Since samples were collected from the same places and the same habitats in this study, the results strongly indicated that there might be some preferences for endophytic fungi of haustorial roots of *T. chinensis* different hosts.

In this study, samples were collected from parasitized branches of seven hosts in the same habitat and during the same season (winter), and the parasitized branches of *D. longan* were more abundant in endophytic fungi. The species and quantity of endophytic fungi in medicinal plants were rich and varied in the different hosts, and the endophytic fungi in medicinal plants are also likely to change with the changes of regions, seasons, growth stage, tissues and organs [37, 38]. Lü et al. (2014) showed that the IR of endophytic fungi in *Atractylodes lancea* of Maoshan type was higher than that in *A. lancea* of Hu Bei type, and the composition of fungal diversity was also partially different [39]. With the change of seasons, the endophytic fungal community showed certain succession rules, and the diversity of endophytic fungi in summer was higher than that in spring and autumn. Shu et al. found that the number and species of endophytic fungi in branches were more than those in the leaves and fruits in different tissues of *Citrus maxima* [40]. Wang et al. showed that the abundance and diversity of fungi in the wild environment were higher than those in a park environment for *Paris polyphyllain* [37, 38]. Ren et al. found that as the altitude increased, the diversity of endophytic fungi in the roots of *Rhododendron simsii* was less varied, the community distribution was more uneven and the dominant fungi were more prominent [41]. In addition, the species of dominant endophytic fungi were geographically different [42, 43]. For example, Xu et al. found that the mycorrhizal fungal community diversity of *Cypripedium tibeticum* (Huanglong Gully, Sichuan) at different altitudes also decreased with an increase of altitude [43]. Therefore, the next step of this research is to explore the gathering of specimens from different habitats and different altitudes, different organizations and different seasons with regards to the endophytic fungi diversity and similarity coefficients of *T. chinensis*, in order to further understand the distribution of these fungi in *T. chinensis*. This will help to explore more endophytic fungi resources, and lay a good theoretical basis for the next steps of antifungal activity screening and in-depth research, with good biocontrol potential.

Endophytic fungi of medicinal plants can inhibit fungi and prevent diseases. They also have a broad spectrum of inhibition properties and have the potential to develop biological control substances [44, 45]. In this work, *N. parvum* and *H. investiens* showed significant antifungal activities against three fungal phytopathogens of medicinal plants. *N. parvum* and *H. investiens* are tall and they are able to occupy space and absorb nutrients rapidly, so as to effectively compete with other pathogens for nutrition and space and inhibit their growth. However, studies have shown that *N. parvum* can cause a variety of woody plants and fruits canker disease and dieback [46–52], which is a harmful pathogenic fungus in agriculture. It has been reported that *N. parvum* had the strongest pathogenicity [53], but in this study, this property it belongs to endophytic fungi. Aly et al. reported that endophytic fungi parasitized on healthy plants can cause plant disease and become pathogenic when they are subjected to environmental stress [54]. Endophytic fungi have a dynamic equilibrium antagonistic relationship with host plants [54]. Whether the fungus causes host disease, its pathogenicity and toxin-producing ability need to be further studied. Therefore, in the selection of biocontrol strains, the endophytic fungi must be tested for the presence of phytotoxins before it can be used.

## Conclusions

This study provides insight for the isolation of endophytic fungal community diversity from seven host plants of *T. chinensis*. The diversity of endophytic fungi in seven host plants of *T. chinensis* was studied for the first time. A total of 150 different endophytic fungi were isolated from the haustorial roots of the seven hosts for the first time. Among of them, *Pestalotiopsis*, *Neopestalotiopsis* and *Diaporthe* were the dominant genera, accounting for 26.67, 17.33 and 31.33% of the total number of strains, respectively. The isolated endophytic fungi (*N. parvum*, *H. investiens* and *Pestalotiopsis* sp.) showed strong inhibitory effects on three pathogens of medicinal plants. At the same time, the crude extracts from the metabolites of these three endophytic fungi also had strong pathogen inhibitory effects. These beneficial microorganisms in our present study will benefit the biological control research and also be a good source of new active compounds. This data also provides a reference for research of other plant species that grow on endophytic microorganisms.

## Materials and methods

### The sample collection of *T. chinensis*

In January 2020, the haustoria of *T. chinensis* from different hosts including *M. alba*, *P. salicina*, *P. chinense*, *D. odorifera*, *B. purpurea*, *D. kaki* and *D. longann* were collected in the *T. chinensis* planting base of Cenxi Funing Village, Wuzhou, Wuzhou City (111°51′14″E, 22°58′12″N) (Fig. 6). The seven different hosts were situated within 20 m of each other. We collected 3–5 haustoria of *T. chinensis* from the same host plants and these were placed in labelled, sealed bags and then they were returned to the laboratory for endophytic fungi isolation within 48 h of collection.

### Isolation and purification of endophytic fungi

Healthy and disease-free haustorial roots of *T. chinensis* from different hosts were selected and the tissues were cut into 5 cm fragments [55]. The surfaces were sterilized with 75% ethanol and 0.1% HgCl_2_ for 2.5 min, and they were then washed with sterile water 3 times. Using sterile forceps and scalpels, they were cut into tissue blocks of about 5 mm in size, and then placed in PDA medium containing streptomycin and plated with 7 blocks per plate. Each sample consisted of 5 plates. The surface sterilization was tested thoroughly by the sterilization method of ultra-clean workbenches, bleach solution and tissue blotting, so as to ensure the accurate separation of endophytic fungi from *T. chinensis* [56]. The cultures were incubated at 28℃ until the mycelia grew from the edges of the tissue blocks, and these were then transferred to PDA plates for culture, purification and preservation. The isolated and purified endophytic fungi were divided into different morphological types according to the characteristics of the culture colonies [26].

### Identification of endophytic fungi

The colony morphology was recorded by referring to Fang’s method [55], and the preliminary identification of colony morphology was conducted by using Wei’s method [57] as well as the use of the international classification website (http://www.indexfungorum.org). For molecular biological identification, a MightyAmp DNA Polymerase Ver.3 (1.25 U/50 µL) kit (Takara Bio Inc., Japan, Cat. No. R076A) was used. The universal primers, ITS1 and ITS4, [58] were used for sequence amplification. Colonies of cultured endophytic fungal hyphae were selected as templates for direct PCR reactions. The target bands were detected by gel imaging methods, and the PCR products of the target bands were sent to BGI (Guangzhou) Co. Ltd. for sequencing. The sequencing results were compared with the sequences in the GenBank of NCBI using BLAST. The linking method of MEGA 7.0 software was used to construct an ITS-rDNA phylogenetic tree by employing the method of Kumar [59]. This allowed the similarity between a target sequence and a phylogenetically relevant referenced 95% sequence to be identified and these were then classified as an unidentified strain. More than 95% of the strains were identified to the genus level and more than 97% were identified to the species level. In addition, it was found that more than 99% were identified as the same species [60].

### Diversity analysis and data processing

Isolation Rate (IR): This refers to the percentage of the number of isolated strains in the sample tissue blocks to the total number of sample tissue blocks, and it was used to measure the abundance of endophytic fungi in plant tissues and the occurrence frequency of multiple infections in each tissue block.

Isolation frequency (IF): This refers to the percentage of the number of isolated endophytic fungi strains to the total number of isolated endophytic fungi, and it was used to reflect the dominance of endophytic fungi of different species (classes) in the total flora. IF ≥ 10% was the dominant genus, IF ≥ 1% was the common genus, IF < 1% are rare genera [61].

Shannon-Wiener (H’) = -Σ(PilnPi), where Pi refers to the percentage of the number of a certain strain of endophytic fungi to the total number of all isolated endophytic fungi, and this was value used to analyze the diversity of endophytic fungi and the complexity of the community in a specific community [62].

Species richness index (Margalef’s index, D): D= (total number of taxa − 1)/ln (total number of individuals), was used to analyze the richness of the taxa [63].

Evenness index (E): E = H’/Ln(S), where H’ is Shannon-Wiener index and S was the number of species found, and this was used to analyze the evenness of the distribution of endophytic fungi of different species in different hosts.

Sorenson’s similarity coefficients (Cs) were used to evaluate the similarity of the species composition of endophytic fungi between two hosts. The formula used to obtain this value was Cs = 2j/(a + b), where j is the number of species shared by the two populations, a is the number of all the species in the first population and b is the number of all the species in the second population.

### In vitro antagonistic assays of endophytes against fungal phytopathogens

The in vitro antagonistic activity of endophytic fungi against *Corynespora cassicola* of *Sarcandra glabra*, *Didymella glomerata* of *Sophora tonkinensis* and *Stagonosporopsis cucurbitacearum* of *Siraitia grosvenorii* were tested using the co-culture method [64, 65]. Briefly, one mycelial plug (6 mm diam) of each 7-d-old fungal phytopathogen was placed in the center of a dish containing approximately 9 mL of PDA, to a final depth of 2 mm. Two mycelial plugs (6 mm diam) from one 7-d-old endophytic fungus were symmetrically placed 2 cm from the endophytic inoculant to establish a co-culture treatment. A sample of only the pathogen was used as a control. Each inhibitory experiment was replicated three times. The cultures were incubated at 28 °C. The radii of the relative fungal phytopathogen colony in the treatment dishes were measured only when the fungal phytopathogen colony had reached the center of the petri dishes in the growth control samples. The average radius of each fungal phytopathogen in the treatment was recorded as R1, and that in the growth control was recorded as R2. The growth inhibition percentage of the fungal phytopathogen with respect to the endophyte, phytopathogen antagonism, was calculated using the following formula:

$$\text{Inhibition\, percentage} \text{(\%)}\text{ }\text{=}\frac{{\text{R}}_{\text{2}}\text{-}{\text{R}}_{\text{1}}}{{\text{R}}_{\text{2}}} \times \text{100}$$

### Crude extract preparation of the fungal fermentation broth

The seed endophytic fungus with the best antibacterial activity was selected as the production strain for small-scale solid fermentation. The production strains were seeded on PDA culture plates and incubated at 28 °C for 5 days. After colony maturation, 5 truffle cakes with a diameter of 6 mm were inoculated into 50 mL triangular vials containing 20 mL of liquid potato medium as seed strains for culture. A total of 2 vials were inoculated and incubated at 28 °C for 3d by shaking at 120 rpm. 60 g of rice was added to a 500mL triangular bottle, 90mL was steamed with water, autoclaved for 20 min, cooled and placed under sterile conditions. 10 mL of seeded bacteria liquid was poured into 500 mL triangular bottles containing solid medium with a total of 4 bottles used. These were mixed well and covered with 8 layers of gauze, and incubate at 28 °C. After 30 days, the gauze was removed and methanol was poured in. The mixture was soaked and fermented and the liquid level was seen to increase by 1-2 cm. This was further soaked for 40 min and then subjected to ultrasonic extraction for 20 min. The mixture was filtered and this procedure was repeated for 3 times. The combined filtrate was kept at 45 °C constant temperature in a water bath and then this was subjected to rotary evaporation in order to dry the extract [66].

### Antibacterial activity of crude extracts of fermentation products

The crude extract from 400 mg of fermentation products was weighed into a 5 mL sterilized centrifuge tube, and 4 mL of methanol was added to completely dissolve it in order to prepare a crude extract solution of 100 mg/mL. This was sterilized by filtration using a Millipore filter (0.22 μm) prior to antimicrobial assays. 1 mL of each solution was absorbed and coated with a pipette gun, and three plates were used for each gradient. The methanol in the plate was evaporated under sterile conditions, and the pathogenic fungi were inoculated into the center of the PDA plate. The cultures were incubated at 28 °C for 5 days, and the growth of the three pathogenic fungi were observed.

### Statistical analysis

Statistical results were expressed as x ± s, with ± being the mean values and s being the standard deviations. SPSS19.0 software was used to conduct univariate ANOVA analysis and the variance homogeneity test for data in each group. P < 0.05 was considered statistically significant.

## Electronic supplementary material

Below is the link to the electronic supplementary material.

**Additional files 1**: Table s1 The isolation rates of endophytic fungi from *T. chinensis* of Fig. 1 raw data; Table s2-s3. The isolation frequency of orders (A) and genera (B) related to *T. chinensis* parasitized on different host plants from the raw data shown in Fig. 3A and B; Table s4. The diversity and distribution of endophytic fungi isolated from *T. chinensis* parasitized on different host plants from raw data in Fig. 4; Table s5. The antifungal activities of crude extracts from fermentation products of endophytic fungi from the raw data of Fig. 6; Table 5

**Additional files 2**: Figure 2 raw data. Phylogenetic tree powerpoint

## Data Availability

Sequences of these twelve strains were submitted to the GenBank database, and the accession numbers obtained were MZ836840, MZ823600, MZ823598, MZ836841, MZ836842, MZ836843, MZ823601, MZ836844, MZ823599, MZ836845, MZ823597 and MZ836846 for strains 1, 15, P6, 20, 17, 24, 4, 13, N6, 22, 31 and 9, respectively. Other data either generated or analyzed during this study are included in the article and its supplementary information files. https://www.ncbi.nlm.nih.gov/search/all/?term=MZ836840;https://www.ncbi.nlm.nih.gov/search/all/?term=MZ823600;https://www.ncbi.nlm.nih.gov/pmc/?term=MZ2823598;https://www.ncbi.nlm.nih.gov/search/all/?term=MZ836841. ;https://www.ncbi.nlm.nih.gov/search/all/?term=MZ836842;https://www.ncbi.nlm.nih.gov/search/all/?term=MZ836843;https://www.ncbi.nlm.nih.gov/search/all/?term=MZ823601;https://www.ncbi.nlm.nih.gov/search/all/?term=MZ836844;https://www.ncbi.nlm.nih.gov/search/all/?term=MZ823599;https://www.ncbi.nlm.nih.gov/search/all/?term=MZ836845;https://www.ncbi.nlm.nih.gov/search/all/?term=MZ823597;https://www.ncbi.nlm.nih.gov/search/all/?term=MZ836846.

## Abbreviations

PCR: Polymerase chain reaction
PDA: Potato Dextrose Agar
PDB: Potato Dextrose Broth
IF: Isolation frequency
IR: Isolation Rate
