# Supplementary material for: Differences and biocontrol potential of haustorial endophytic fungi from Taxillus Chinensis on different host plants
Source: BMC Microbiol. 2023 May 13;23:128. doi: 10.1186/s12866-023-02878-x (PMC10182615; doi:10.1186/s12866-023-02878-x)
Supplement: Supplementary file 2 — Additional files 2: Figure 2 raw data. Phylogenetic tree powerpoint [file 12866_2023_2878_MOESM2_ESM.pptx]

## Slide 1
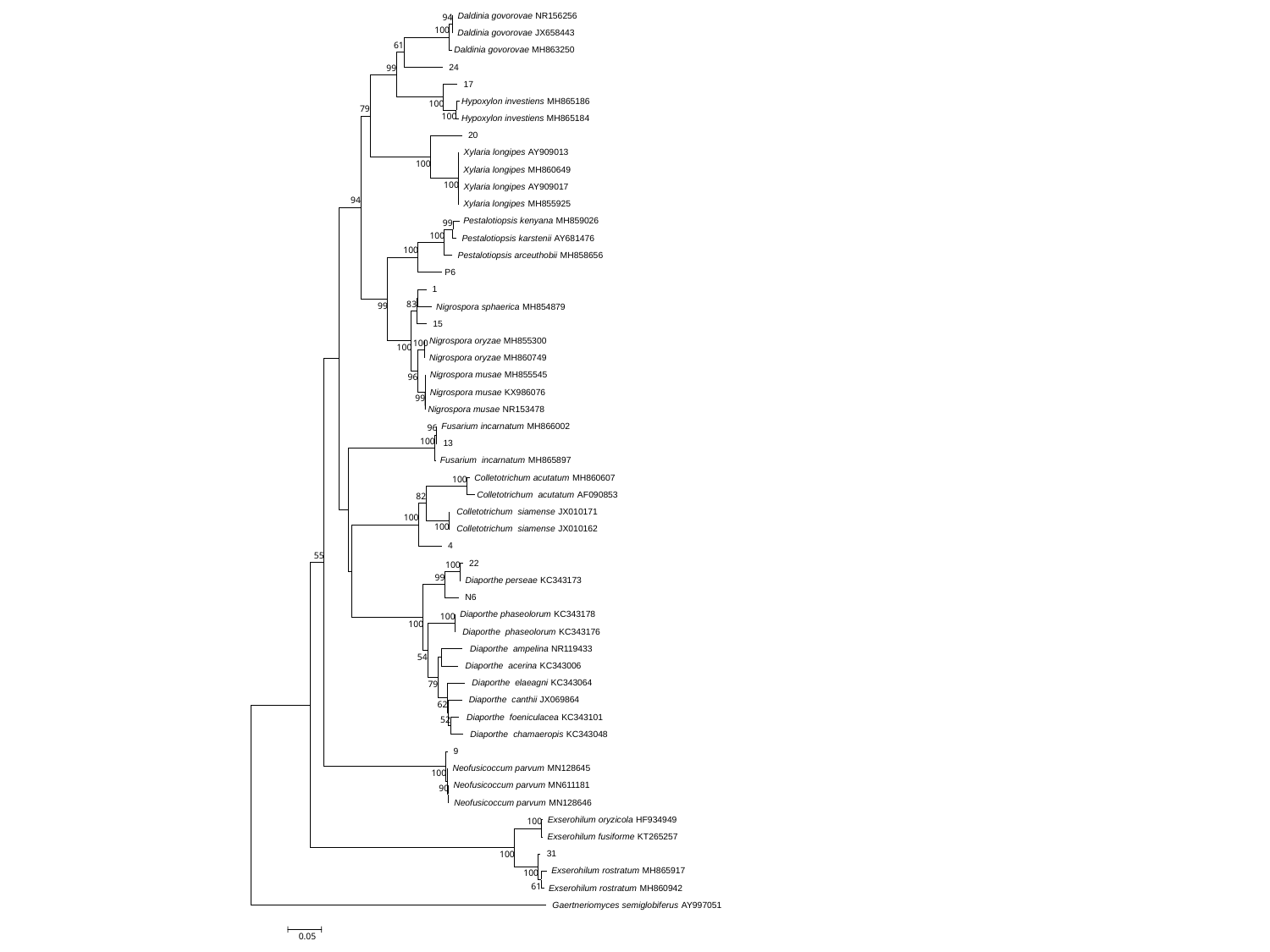

Daldinia govorovae NR156256
94
100
 Daldinia govorovae JX658443
61
Daldinia govorovae MH863250
 24
99
 17
Hypoxylon investiens MH865186
100
79
100
Hypoxylon investiens MH865184
 20
 Xylaria longipes AY909013
100
 Xylaria longipes MH860649
100
 Xylaria longipes AY909017
94
 Xylaria longipes MH855925
 Pestalotiopsis kenyana MH859026
99
100
 Pestalotiopsis karstenii AY681476
100
 Pestalotiopsis arceuthobii MH858656
 P6
 1
83
99
 Nigrospora sphaerica MH854879
 15
 Nigrospora oryzae MH855300
100
 Nigrospora oryzae MH860749
 Nigrospora musae MH855545
 Nigrospora musae KX986076
 Nigrospora musae NR153478
 Fusarium incarnatum MH866002
96
100
 13
 Fusarium incarnatum MH865897
 Colletotrichum acutatum MH860607
100
 Colletotrichum acutatum AF090853
82
 Colletotrichum siamense JX010171
100
100
 Colletotrichum siamense JX010162
 4
55
 22
100
99
 Diaporthe perseae KC343173
 N6
 Diaporthe phaseolorum KC343178
100
100
 Diaporthe phaseolorum KC343176
 Diaporthe ampelina NR119433
54
 Diaporthe acerina KC343006
 Diaporthe elaeagni KC343064
79
 Diaporthe canthii JX069864
62
 Diaporthe foeniculacea KC343101
52
 Diaporthe chamaeropis KC343048
 9
 Neofusicoccum parvum MN128645
100
 Neofusicoccum parvum MN611181
90
 Neofusicoccum parvum MN128646
 Exserohilum oryzicola HF934949
100
 Exserohilum fusiforme KT265257
 31
100
 Exserohilum rostratum MH865917
100
61
 Exserohilum rostratum MH860942
 Gaertneriomyces semiglobiferus AY997051
100
96
99
0.05
